# Four-year course of quality of life and obsessive–compulsive disorder

**DOI:** 10.1007/s00127-019-01779-7

**Published:** 2019-09-20

**Authors:** Karin C. P. Remmerswaal, Neeltje M. Batelaan, Adriaan W. Hoogendoorn, Nic J. A. van der Wee, Patricia van Oppen, Anton J. L. M. van Balkom

**Affiliations:** 1 Department of Psychiatry, Amsterdam UMC, Vrije Universiteit, Amsterdam Public Health Institute and GGZ inGeest Specialized Mental Health Care, Amstelveenseweg 589, 1081 JC Amsterdam, The Netherlands; 2 Department of Psychiatry, Leiden University Medical Centre, Leiden, The Netherlands

**Keywords:** OCD, Obsessive–compulsive disorder, QoL, Quality of life, Longitudinal

## Abstract

**Objective:**

Patients with obsessive compulsive disorder (OCD) have high disease burden. It is important to restore quality of life (QoL) in treatment, so that patients become able to live a fulfilling life. Little is known about the longitudinal course of QoL in patients with OCD, its association with remission from OCD, and about factors that contribute to an unfavourable course of QoL in remitting patients.

**Methods:**

Study on the 4-year course of QoL of patients with chronic (*n *= 144), intermittent (*n *= 22), and remitting OCD (*n *= 73) using longitudinal data of the Netherlands Obsessive Compulsive Disorder Association (NOCDA; complete data: *n *= 239; imputed data *n *= 382). The EuroQol five-dimensional questionnaire (EQ-5D) utility score was used to assess QoL. In patients with remitting OCD, we examined patient characteristics that contributed to an unfavourable course of QoL, including sociodemographics, OCD characteristics, psychiatric comorbidity, and personality traits.

**Results:**

Course of QoL was associated with course of OCD. QoL improved in those who remitted from OCD; however, even in these patients, QoL remained significantly below the population norms. The correlation between QoL and severity of OCD was only moderate: *r *= − 0.40 indicating that other factors besides OCD severity contribute to QoL. In remitters, more severe anxiety and depression symptoms were related to a lower QoL. Results were similar in complete and imputed data sets.

**Conclusions:**

Remission from OCD is associated with improvement of QoL, but comorbid anxiety and depression symptoms hamper the improvement of QoL. QoL could be improved by reducing OCD symptoms in patients with OCD and by treating comorbid anxiety and depression symptoms in remitting patients.

**Electronic supplementary material:**

The online version of this article (10.1007/s00127-019-01779-7) contains supplementary material, which is available to authorized users.

## Introduction

Patients with mental disorders like schizophrenia, major depressive disorder, anxiety disorders, and obsessive compulsive disorder (OCD) experience an impaired Quality of Life (QoL) compared with the general population. The WHO defines QoL as ‘an individual’s perception of their position in life in the context of the culture and value systems in which they live and in relation to their goals, expectations, standards, and concerns’ (https://www.who.int/healthinfo/survey/whoqol-qualityoflife; page 1). Thus, QoL is a complex concept, dependent of the ability of a person to function in life domains such as his/her physical health, psychological state, and social relationships [1]. It is well known that mental disorders impact on the ability to function in these life domains and, hence, have a strong impact on QoL.

The relationship between psychopathology and QoL, however, is complex. In particular following successful treatment of the index mental disorder, QoL is often not restored to the level of the general population, which implies that remitted patients still may experience problems in their daily physical, psychological, and social functioning [2, 3]. For example, in patients with schizophrenia, it was found that 3 months to 2 years following successful treatment QoL was either improved [4, 5] or had not changed [6–8]. Fluctuations in QoL in schizophrenia appeared to depend more on severity of comorbid depressive and anxiety symptoms and psychosocial factors than on presence and severity of psychotic symptoms [4, 9], indicating that factors other than the severity of the disorder itself impact on QoL. Likewise, in patients with major depressive disorder and anxiety disorders, QoL improved after treatment, but levels of QoL remained below population norms, even when patients were remitted [3, 10–13].

OCD is a mental disorder with a tremendous impact on QoL, underlining the need to fully understand the relationship between OCD and QoL [14, 15]. In the same vein as schizophrenia, anxiety, and depressive disorders, generally, it was shown that QoL was not fully restored following successful treatment [16]. For example, after treatment with eighteen sessions of exposure in vivo with response prevention, OCD symptom severity and QoL improved significantly although QoL remained below community norms [17, 18]. Likewise, in a randomized-controlled trial comparing the effect of SRI augmentation with 8 weeks of either exposure in vivo with response prevention, risperidone or pill-placebo, QoL improved significantly only in the exposure and response prevention condition; however, it did not reach the level of the general population, even though OCD symptom severity decreased significantly in all conditions [19]. In contrast, QoL did improve up till the level of the general population—and OCD symptom severity decreased—in a study in which patients received escitalopram for 16 weeks [20].

Although such short-term outcomes are informative to evaluate direct treatment effects, changes in QoL may require more time. For example, social functioning requires to make contact with other people and to build a social network. Moreover, sustained outcomes may be more important than short-term successes. Data from studies with longer timeframes suggest that over time, OCD severity further decreases and QoL further improves, but also after longer follow-up periods, QoL still remains significantly impaired compared to that of healthy controls. For example, in long-term pharmacological treatment studies, it was found that OCD severity and QoL improved after 24–52 weeks of treatment with, respectively, fluvoxamine, fluoxetine, escitalopram, or paroxetine [2, 21, 22]. Albeit improved, in all these three studies, QoL at follow-up remained below community norms. In addition, most aspects of QoL, except social QoL, were significantly improved after 12 months of multimodal inpatient treatment, mainly consisting of exposure in vivo with response prevention but, also in this study, QoL remained below population norms [23]. Finally, after 3–5 years of treatment with deep brain stimulation, severity of OCD and QoL were improved, except social QoL, but QoL remained below community norms [24].

The general finding that, both in short-term and in longer term studies, OCD symptom reduction is not equated with regaining QoL suggests that factors other than OCD impact on QoL. Indeed, various longitudinal treatment studies indicate that OCD symptom reduction is not or only weakly related to QoL improvement [16, 21, 24] and to improvement of depressive symptoms [23]. Cross-sectional studies examining the factors associated with QoL in OCD report a variety of factors to impact on QoL in OCD patients, including older age, female sex, unemployment, contamination, hoarding and symmetry symptom dimensions, over-responsibility for harm, comorbid anxiety and depression symptoms, low social status, and perceived low social support [15, 16, 25–30]. However, due to the cross-sectional design of these studies, causal inferences cannot be made.

To get a clear understanding of the long-term course of QoL and its association with OCD, long follow-up periods are needed given the chronic course of OCD and the time required for changes in QoL. A longitudinal design is also required to obtain a clearer view on the factors associated with an unfavourable course of QoL in remitting patients. Malleable predictors may personalize treatment and improve outcome of QoL in patients with OCD.

The goal of the present study was (1) to explore the 4-year course of QoL in a large, representative cohort of patients with OCD, (2) to investigate the association between course of QoL and course of OCD, and (3) to identify predictors of an unfavourable course of QoL in patients with remitting OCD. For this study, we have selected predictors that have been associated with QoL in OCD or with course and severity of OCD, such as sociodemographic variables, clinical variables, personality traits [31, 32], and the quality of the support from the social network [33], which is also determined by need for affiliation and attachment style of the patient [34, 35]. We hypothesized that: (1) QoL improves over time, but remains lower than the QoL of the general population even after 4 years; (2) QoL and OCD are weakly correlated; (3) course of QoL is dependent on various factors other than OCD.

## Materials and methods

### Procedure

Data were derived from The Netherlands Obsessive Compulsive Disorder Association (NOCDA) study, an ongoing longitudinal cohort study investigating the naturalistic long-term course of OCD in patients referred to mental health care centres and to examine determinants in predicting the course of OCD. The NOCDA study design and baseline characteristics of the study sample are described in detail elsewhere [36]. The NOCDA study was accredited by the Medical Ethical Committee of the VU-university Medical Centre in 2005.

After intake at one of the contributing mental health clinics, 687 patients aged 18 years and over with a lifetime diagnosis of OCD, as determined by the administration of the Structured Clinical Interview for DSM-IV Axis I Disorders (SCID-I) [37], were asked to participate in the NOCDA study. Since NOCDA aims to follow a large representative sample of OCD subjects in different stages of the disease and with different degrees of illness severity, the only exclusion criterion was an inadequate understanding of the Dutch language for the purposes of the completion of interviews and self-report questionnaires. Comprehensive measurements were done at baseline and after 2 and 4 years.

Of the 687 patients who were asked to participate in the NOCDA study, 419 (60.9%) gave written informed consent and were enrolled in the study. A comparison on basic demographic characteristics between patients that did *(n *= 419) and did not (*n *= 268) agree to participate yielded no significant differences.

Baseline measurements took place between 2005 and 2009, and included validated semi-structured interviews and self-report questionnaires to gather information on a broad range of variables related to (amongst others) OCD, comorbidity, and psychosocial consequences. The baseline assessment took about 5 h. All included participants were contacted after 2 years and 4 years for follow-up, irrespectively of their treatment status. The 2-year and 4-year assessments took about 3 h, and in most cases (80%), they were done by the same research assistant. During the follow-up period, participants received treatment as usual that was based on Dutch multidisciplinary guidelines.

### Participants in the present study

In the present study, only those patients who had a current OCD diagnosis at baseline were included, pertaining to 382 patients at baseline, 278 patients at 2-year follow-up (total dropout 27%), and 268 patients at 4-year follow-up (total dropout 30%). Of 239 patients, complete SCID data at baseline, 2, and 4 years were available. Patients with incomplete data did not differ significantly from those with complete data on sociodemographic and clinical characteristics except that they were younger (*F*(1,  380) = 4.16; *p *= 0.04) and less educated (*F*(1, 380) = 23.17; *p *< 0.01).

Patients were divided in three groups: chronic (current diagnosis of OCD at baseline, 2- and 4-year follow-up; *n *= 144), intermittent (current diagnosis of OCD at baseline and 4-year follow-up but not at 2-year follow-up; *n *= 22), and remitting (current diagnosis of OCD at baseline but not at 4-year follow-up, irrespective of diagnostic status at 2-year follow-up; *n *= 73). This study is reported conform the STROBE statement [38].

### Primary outcome measure: QoL

The self-rated *EuroQol five-dimensional questionnaire (EQ*-*5D)* was used to assess QoL. It is a widely used, generic QoL instrument. It is applicable in many populations and can be used to compare QoL in various conditions. The EQ-5D contains five dimensions significant for QoL: mobility, self-care, daily activities, pain/discomfort, and depression/anxiety. Each dimension is rated at three levels: no problems, some problems, and major problems. These health states are converted into an index score—the EQ-5D—reflecting the generic overall QoL. The EQ-5D has a value between 1 (best possible health) and 0 (worst possible health). The EQ-5D was proven reliable, valid, and feasible [39–43].

### Potential predictors of course of QoL in remitting patients

Repeatedly measured variables included severity of OCD, number of current comorbid mental disorders, comorbid anxiety and depressive symptoms, loneliness, need for affiliation, social support, and social network. These were assessed at baseline, 2- and 4-year follow-up. The variable time of remission was determined on the basis of the time-dependent variable current diagnosis of OCD, but is not itself a time-dependent variable. All other characteristics were assessed at baseline only: sociodemographics, age of onset, personality characteristics, attachment style, and perceived expressed emotion.

Sociodemographic characteristics included age (in years), gender, partner (yes, no), children (yes, no), education (number of years), and employment (yes, no).

The severity of OCD was assessed by the *Yale Brown Obsessive Compulsive Scale for Severity (Y*-*BOCS)* [44, 45]. The interrater reliability (ICC = 0.96) and test–retest reliability (ICC = 0.85) of the Y-BOCS are high [46].

*Age of onset of OCD* was assessed with the SCID-I as the earliest age at which patients fulfilled the criteria for OCD. In case of remission, *time of remission* was defined as early (remission at 2-year follow-up) or late (remission at 4-year follow-up). To assess the *number of current comorbid mental disorders*, the ascertained diagnoses on the SCID-I were counted. Interrater reliability of the SCID-I is fair-to-excellent, and the test–retest reliability and validity are substantial [47, 48]. Comorbid depressive symptoms were measured by the *Beck Depression Inventory (BDI)* [49, 50]. Comorbid anxiety symptoms were assessed by the *Beck Anxiety Inventory (BAI)* [51].

Furthermore, personality characteristics according to the Big Five were assessed with the *Five*-*Factor Personality Inventory (FFPI)* [52]. Subscales of the FFPI are: extraversion, agreeableness, conscientiousness, emotional stability, and autonomy. Attachment style was assessed with the G*eneral Attachment Style Questionnaire* [53], with the subscales: dismissing, preoccupied, fearful, and secure. Loneliness was assessed with the *Loneliness Scale* [54], subscales: emotional loneliness and social loneliness. The need for affiliation was assessed with the *Need for Affiliation Scale* [35]. Social support was assessed with the *Social Support Inventory* [55]. Subscales of the SSI are: emotional support, informative support, social companionship, and instrumental support. Perceived expressed emotion of significant others was assessed with the *Level of Expressed Emotion (LEE)* [56]. Subscales of the LEE are: lack of emotional support, perceived intrusiveness, perceived irritation, and perceived criticism. Social network (number of friends) was assessed by an interview designed for the NOCDA study.

### Quality aspects of NOCDA

The NOCDA study was coordinated by the academic department at VU Medical Centre/GGZ inGeest Amsterdam and included seven sites that were specialized OCD mental health clinics spread over the Netherlands. All research assistants had extensive experience with the assessment of OCD. In addition, they received a 2-day course, and regular follow-up 1-day training sessions in which videos of the SCID were rated, assessor rating scales were practiced and questions and problems raised by the research assistants could be addressed. The first two interviews of all research assistants were audiotaped and monitored by the fieldwork coordinator to address any misunderstandings or errors in performing the measurements. All subsequent interviews were audiotaped for future reference. The monitoring of these audiotapes was continuously performed randomly on about 10% of all taped interviews, as well as on the basis of questions raised by the research assistants and the fieldwork coordinator. Assessments were done by around 30 research assistants (profession: psychologist or research nurse).

### Power considerations

Differences in QoL between patient groups versus the general population will be evaluated in terms of between-group effect sizes. We expect that the between-group effect sizes will range from medium, i.e., Cohen’s *d *= 0.5, when comparing patients with severe complaints to the general population, to small, i.e., *d *= 0.2, when comparing patients with mild severity to the general population. Differences in mean QoL scores of patient groups and the general population will be tested using one-sample *t* tests. Assuming a total sample size of *n *= 382, the minimal detectable effect size will be (Cohen’s) *d *= 0.15 for the total patient group and *d *= 0.19, *d *= 0.27 and *d *= 0.47 for patient subgroups that consist of 60%, 30%, and 10% of the total group, respectively. Restricting the sample to the 239 respondents with complete cases inflates minimal detectable effect sizes slightly to *d *= 0.18 for the total sample and *d *= 0.24, *d *= 0.33, and *d *= 0.59 for patient subgroups mentioned earlier.

### Statistical analyses

Baseline characteristics of the total sample were summarized. Next, baseline characteristics of the three patient groups were compared using one-way ANOVAs for continuous variables and Chi-square statistics for categorical variables. Post hoc tests consisted of pairwise *t* tests with Bonferroni correction for continuous variables and column proportions *z* tests with Bonferroni correction for categorical variables.

Furthermore, the mean QoL of the total sample and the patient groups were compared to the mean QoL of the general Dutch population with one-sample *t* tests. We used data from Szende et al. [57] to determine the mean EQ-5D of the general Dutch population, which is 0.89. Effect sizes (Cohen’s *d*) within time and between groups were calculated using pooled standard deviations assuming SD = 0.20 for the general population. A Cohen’s *d* of 0.2 is indicative of a small-effect size, *d *= 0.5 of a medium-effect size, and *d *= 0.8 of a large-effect size. The correlation between QoL and Y-BOCS total score over all measurements was established with Pearson’s correlation coefficient *r*.

We examined the 4-year course of QoL using linear mixed models (LMM). LMM was used to correct for the correlation in the data due to the repeated measure design. In a second analysis, we examined the association between course of QoL and course of OCD. In this LMM analysis, time and group (remitting, intermittent, and chronic) were added as categorical variables. To examine whether course of QoL differed for the three groups, the group-by-time interaction terms were entered in the model. Effect sizes between group and time were calculated using pooled baseline standard deviations.

In a third analysis (using only respondents with remitting OCD), we examined whether change in QoL was associated with the possible predictor variables, using LMM’s allowing quadratic development over time. Next, all possible predictor variables were added to the basic model, one at a time. Y-BOCS, number of disorders, BAI, BDI, social network, loneliness (emotional and social), need for affiliation, and all subscales of SSI were treated as time-dependent (repeatedly measured) variables. All other variables were treated as time-independent (baseline only) variables. For time-dependent variables, it was investigated whether a random slope improved the model. Thereafter, multivariable analyses were conducted in four steps. In step one, all sociodemographic variables showing statistical significance (*p* < 0.05) in the univariable analyses were analysed together (model one). Model 2 included all clinical variables showing statistical significance in the univariable analyses and model 3 included all psychosocial variables showing statistical significance in the univariable analyses. The final model (model 4) included all variables showing statistical significance (*p* < 0.05) in model 1–3. We regarded correlations of 0.80 and above as a sign of multicollinearity [58].

First, statistical analyses were conducted on the complete data set, including 239 participants. Next, using multiple imputation techniques, a second data set was created (*n *= 382) allowing to investigate potential bias due to missing data. We describe the incompleteness of the data for variables at baseline, 2-year and 4-year follow-up separately (see online supplement). The appropriateness of the imputation method relies on the Missing at Random (MAR) assumption, which allows the missingness of data to depend on the observed variables. We applied MI by chained equations (MICE) using predictive mean matching with a single nearest neighbour for all variables used in the analyses to create 100 imputed datasets.

Data analysis was performed with IBM SPSS Statistics version 25 [59]. Multiple imputations and analyses on the multiple imputation data set were performed with Stata version 15.1 [60]. Since the analyses with complete data (*n *= 239) and imputed data (*n *= 382) yielded identical outcomes except for two minor results, we only report the complete data analyses here. Results that differed will be indicated.

## Results

### Sample characteristics at baseline

In Table 1, the characteristics at baseline of the total sample (*n *= 239) and the three OCD groups are presented. Patients with remitting OCD (*n *= 73) received significantly more education,^1^ had more frequently employment and had significantly less severe OCD symptoms compared to patients with chronic OCD (*n *= 144). Patients with intermittent OCD (*n *= 22) scored in between. Groups did not differ significantly on psychosocial variables including attachment style, personality characteristics, perceived expressed emotion, social network, loneliness, need for affiliation, and social support.

**Table 1:** Baseline characteristics of OCD patients

	Total sample Mean (sd) or % *n *= 239	*n*	Chronic (1) Mean (sd) or % *n *= 144	Intermittent (2) Mean (sd) or % *n *= 22	Remitting (3) Mean (sd) or % *n *= 73	Test statistic	*p* value	Post hoc analysis *p *< 0.05
Sociodemographics								
Age (years)	37.3 (10.9)	239	38.0 (11.0)	37.9 (13.4)	35.7 (10.0)	*F*(2, 236) = 1.10	0.34	
Gender, female	54%	239	58%	50%	45%	*X*^2^(2) = 3.48	0.18	
Partner, yes	64%	234	61%	77%	66%	*X*^2^(2) = 2.39	0.30	
Children, yes	38%	239	38%	36%	38%	*X*^2^(2) = 0.03	0.99	
Education (years)	13.1 (3.1)	239	12.6 (3.1)	13.3 (3.4)	13.9 (3.1)	*F*(2, 236) = 4.18	0.02*	1 < 3
Employment, yes	55%	239	47%	55%	71%	*X*^2^(2) = 11.94	<0.01*	1 < 3
Clinical characteristics								
Y-BOCS total	20.8 (7.1)	236	22.3 (6.8)	19.1 (6.0)	18.3 (7.3)	*F*(2, 233) = 8.84	<0.01*	1 > 3
Late age of onset OCD^a^, yes	36%	217	30%	41%	47%	*X*^2^(2) = 5.73	0.06	
Number of disorders^b^	1.9 (1.1)	239	1.9 (1.2)	1.8 (0.8)	1.9 (1.0)	*F*(2, 236) = 0.23	0.80	
Beck anxiety index	17.4 (11.3)	230	17.8 (11.6)	16.5 (8.9)	17.0 (11.5)	*F*(2, 227) = 0.21	0.81	
Beck depression inventory	15.6 (9.7)	227	16.2 (9.8)	14.9 (8.9)	14.7 (9.7)	*F*(2, 224) = 0.63	0.54	

^a^Onset ≥ 20 years

^b^Number of current comorbid psychiatric disorders

*p < 0.05

### Four-year course of QoL in patients with OCD

Table 2 presents the 4-year course of QoL of the total sample and the comparisons with the general population. At all measurements, one-sample *t* tests indicated that the mean QoL of the total sample was significantly lower than the mean QoL of the general population. QoL improved significantly from baseline to 2-year follow-up [*β*(S.E.) = 0.082 (0.016), *p *< 0.01, 95% CI = (0.050, 0.114)], but did not change critically from 2- to 4-year follow-up [*β*(S.E.) = − 0.025 (0.016), *p *= 0.13, 95% CI = (− 0.057, 0.007)]. Correlation (Pearson’s *r*) between QoL and Y-BOCS total score over all measurements was − 0.40, which is a modest correlation [58].

**Table 2:** Four-year course of QoL (EQ-5D) of patients with OCD and comparison with QoL of the general population with one-sample *t* tests^a^ and within-time between-group effect sizes of OCD group versus general population^b^

EQ-5D	Baseline Mean (SD) *n* *t*(*df*) *p* ES	2-Year follow-up Mean (SD) *n* *t*(*df*) *p* ES	4-Year follow-up Mean (SD) *n* *t*(*df*) *p* ES
Total sample	0.67 (0.28) 228 *t*(227) = − 11.80 < 0.01*	0.75 (0.25) 215 *t*(214) = − 8.09 < 0.01*	0.73 (0.25) 220 *t*(219) = − 9.62 < 0.01*
	0.92	0.58	0.67
Chronic OCD	0.65 (0.28) 137 *t*(136) = − 10.05 <0.01*	0.70 (0.24) 125 *t*(124) = − 8.93 <0.01*	0.66 (0.25) 129 *t*(128) = − 10.37 <0.01*
	1.00	0.79	0.96
Intermittent OCD	0.75 (0.21) 22 *t*(21) = − 3.20 < 0.01*	0.85 (0.18) 21 *t*(20) = − 1.07 0.30	0.81 (0.14) 22 *t*(21) = − 2.74 0.01*
	0.68	0.20	0.39
Remitting OCD	0.69 (0.30) 69 *t*(68) = − 5.62 < 0.01*	0.83 (0.24) 69 *t*(68) = − 2.02 0.05*	0.83 (0.24) 69 *t*(68) = − 2.20 0.03*
	0.80	0.24	0.24

^a^One-sample *t* test against test value 0.89

^b^ES = within-time between-group effect size of OCD group versus general population (Cohen’s *d*), obtained using pooled standard deviations assuming SD = 0.20 for the general population

*p < 0.05

### Four-year course of QoL and the association with course of OCD

**Fig. 1:**
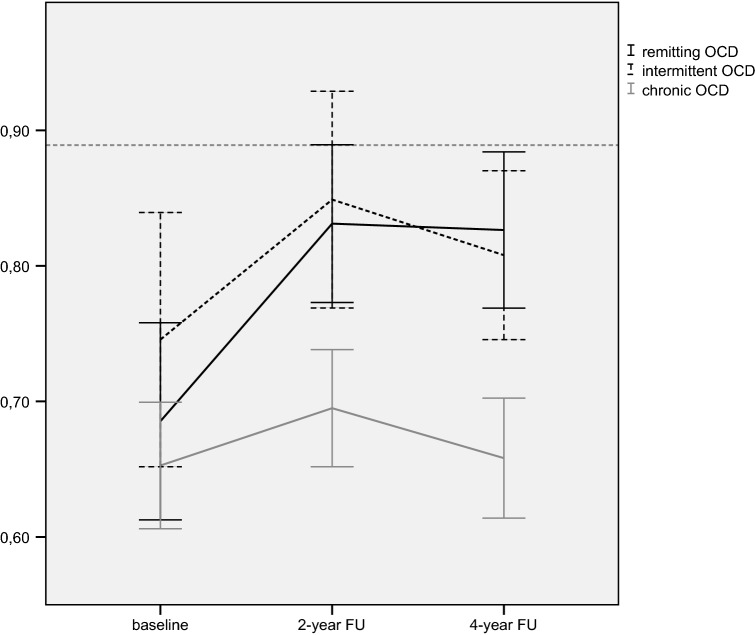
Four-year course of QoL of patients with chronic, intermittent, and remitting OCD. QoL of patients with remitting OCD improved significantly more than patients with chronic OCD from baseline to 2-year follow-up [*β*(S.E.) = 0.110 (0.036), *p *< 0.01]. QoL of patients with chronic OCD was significantly worse than the other two groups on average over time [chronic versus remitting: (*β*(S.E.) = − 0.114 (0.031), *p *< 0.01; chronic versus intermittent: (*β*(S.E.) = − 0.128 (0.049), *p *= 0.01]. The reference line displays the QoL of the general population (0.89)^45^

Table 2 and Fig. 1 present the 4-year course of QoL in chronic, intermittent, and remitting OCD and the comparison with the mean QoL of the general population. At baseline, there was no significant difference in QoL between the patient groups (*F*(2, 225) = 1.17, *p *= 0.31). At all assessments, the mean QoL of the patient groups remained significantly below the mean QoL of the general population, except QoL of patients with intermittent OCD at 2-year follow-up, which did not significantly differ from the QoL of the general population. The effect sizes of the difference in QoL of the total sample of patients with the general Dutch population were large (Cohen’s *d* between 0.67 and 0.92), indicating that QoL of patients with OCD differs largely from the general population.

Table 3 presents the results of the LMM analyses, investigating the association between course of QoL and course of OCD. The data suggest that course of QoL is significantly associated with course of OCD: QoL of patients with remitting OCD improved significantly more than patients with chronic OCD from baseline to 2-year follow-up but not from 2- to 4-year follow-up. The effect sizes are moderate (Cohen’s *d *= − 0.38) and small (Cohen’s *d *= − 0.14), respectively, indicating that there is a moderate difference in the improvement of QoL of patients with remitting OCD versus patients with chronic OCD from baseline to 2-year follow-up and a small difference from 2- to 4-year follow-up. In addition, there was a significant difference between the QoL of patients with chronic OCD and the other two groups on average over time [remitting: (*β*(S.E.) = 0.114 (0.031), *p *< 0.01, 95% CI = (0.052, 0.175); intermittent: (*β*(S.E.) = 0.128 (0.049), *p *= 0.01, 95% CI = (0.031, 0.225)].

**Table 3:** Results of LMM analysis of the 4-year course of QoL (EQ-5D) of patients with chronic, intermittent, and remitting OCD

OCD group comparison	From baseline to 2-year follow-up *β* (S.E.) *p* 95% CI ES^a^	From 2- to 4-year follow-up *β* (S.E.) *p* 95% CI ES
Chronic vs intermittent patients	− 0.056 (0.055) 0.31 (− 0.165, 0.052) − 0.23	0.002 (0.055) 0.97 (− 0.106, 0.110) 0.01
Chronic vs remitting patients	− 0.110 (0.036) < 0.01* (− 0.180, − 0.041) − 0.38	− 0.030 (0.036) 0.40 (− 0.099, 0.040) − 0.14
Intermittent vs remitting patients	− 0.054 (0.058) 0.36 (− 0.169, 0.061)	− 0.032 (0.058) 0.59 (− 0.146, 0.083)
	− 0.21	− 0.15

^a^ES = between-group between-time effect sizes (standardized using pooled start-time standard deviations)

*p < 0.05

### Predictors of an unfavourable course in remitters from OCD

Table 4 shows the results of the analyses that investigated whether change in QoL in remitters from OCD was associated with sociodemographic, clinical, and psychosocial variables. In the univariable analyses, unemployment, late (vs early) remission, less emotional stability (time-independent variables) and more severe OCD, more comorbid mental disorders, more severe comorbid anxiety and depression, a smaller social network, more emotional loneliness and more social loneliness, less emotional support, and less social companionship (time-dependent variables) were all significantly associated with an unfavourable 4-year course of QoL in remitters from OCD.^2^ In the final multivariate model, only more severe comorbid anxiety and depression symptoms remained significantly related to an unfavourable course of QoL, suggesting that comorbid anxiety and depression are most strongly associated with course of QoL.

**Table 4:** Predictors of the 4-year course of QoL in remitters from OCD, *n *= 73

	Bivariate		Model 1^a^		Model 2^b^		Model 3^c^		Model 4^d^	
	*β* (S.E.)	*p*	*β* (S.E.)	*p*	*β* (S.E.)	*p*	*β* (S.E.)	*p*	*β* (S.E.)	*p*
Sociodemographics										
Age (years)	− 0.002 (0.003)	0.38								
Gender, female	0.016 (0.055)	0.78								
Partner, yes	− 0.050 (0.059)	0.40								
Children, yes	− 0.016 (0.057)	0.77								
Education (years)	0.010 (0.009)	0.27								
Employment, yes	0.126 (0.059)	0.04*	0.126 (0.059)	0.04*					0.038 (0.041)	0.36
Clinical characteristics										
Y-BOCS^e^	− 0.008 (0.002)	< 0.01*			− 0.001 (0.002)	0.67				
Age of onset OCD^f^, late	− 0.017 (0.058)	0.78								
Time of remission^g^, late	− 0.122 (0.054)	0.03*			− 0.034 (0.037)	0.36				
Number of disorders^e,h^	− 0.087 (0.017)	< 0.01*			− 0.024 (0.017)	0.17				
Beck Anxiety Index^e^	− 0.012 (0.002)	< 0.01*			− 0.005 (0.002)	< 0.01*			− 0.005 (0.002)	< 0.01*
Beck Depression Inventory^e^	− 0.019 (0.002)	< 0.01*			− 0.014 (0.002)	< 0.01*			− 0.015 (0.002)	< 0.01*
Psychosocial variables										
Attachment style: dismissing	0.021 (0.014)	0.13								
Attachment style: preoccupied	− 0.007 (0.013)	0.62								
Attachment style: fearful	− 0.018 (0.013)	0.19								
Attachment style: secure	0.010 (0.015)	0.51								
FFPI^i^ extraversion	0.021 (0.027)	0.44								
FFPI agreeableness	0.002 (0.025)	0.93								
FFPI conscientiousness	0.017 (0.024)	0.48								
FFPI emotional stability	0.076 (0.019)	< 0.01*					0.066 (0.019)	< 0.01*	0.011 (0.015)	0.46
FFPI autonomy	0.030 (0.025)	0.24								
LEE^j^ lack of emotional support	0.000 (0.003)	0.92								
LEE perceived intrusiveness	− 0.007 (0.005)	0.21								
LEE perceived irritation	− 0.001 (0.007)	0.88								
LEE perceived criticism	− 0.007 (0.012)	0.54								
Social network^e^	0.009 (0.004)	0.03*					0.007 (0.004)	0.08		
Loneliness emotional^e^	− 0.031 (0.008)	< 0.01*					− 0.013 (0.011)	0.25		
Loneliness social^e^	− 0.025 (0.010)	0.02*					− 0.008 (0.012)	0.50		
Need for affiliation^e^	0.007 (0.010)	0.51								
SSI^k^ emotional support^e^	0.016 (0.007)	0.02*					0.001 (0.009)	0.92		
SSI informative support^e^	0.015 (0.008)	0.06								
SSI social companionship^e^	0.013 (0.006)	0.04*					0.001 (0.008)	0.91		
SSI instrumental support^e^	0.011 (0.007)	0.12								

^a^All sociodemographic variables showing statistical significance (*p* < 0.05) in the univariable analyses analysed together

^b^All clinical variables showing statistical significance (*p* < 0.05) in the univariable analyses analysed together

^c^All psychosocial variables showing statistical significance (*p* < 0.05) in the univariable analyses analysed together

^d^All variables showing statistical significance (*p* < 0.05) in model 1–3 analysed together

^e^Time-dependent variable (repeatedly measured)

^f^Late onset ≥ 20 years

^g^Late remission (at 4-year follow-up; *n *= 31) vs early remission (at 2-year follow-up; *n *= 42)

^h^Number of current comorbid psychiatric disorders

^i^Five-factor personality inventory

^j^Level of expressed emotion

^k^Social support inventory

*p < 0.05

### Results using multiple imputed data

The results obtained using multiple imputed data are deferred to Electronic supplement material, since they are very similar to the results above that were obtained from complete cases. Missing information was mostly due to drop out. The variables with missing values are displayed in Electronic supplement material also. Although information on diagnosis was always available, some missing values occurred in demographic variables [education (1 missing value), having a partner (9 missing values) and clinical measures (20 up to 30 missing values; the maximum number of 39 missing values for age of onset)]. In total, 104 patients missed the 2-year follow-up, while 114 patients missed the 4-year follow-up interview. The missing data pattern was that 239 patients assessed all interviews, 29 patients missed the 2-year follow-up interview only, and 39 patients missed the 4-year follow-up interview only, while 75 patients missed both follow-up interviews.

## Discussion

The present study underlines the importance of assessing QoL in patients with OCD. We have investigated the 4-year course of QoL, its association with remission from OCD and the factors that contribute to an unfavourable course of QoL in remitting OCD patients.

The mean QoL of the total sample of patients with OCD of our study improved from baseline to 2-year follow-up and this was maintained at 4-year follow-up, but it remained substantially lower than the QoL of the general population, which is congruent with previous studies [16, 21, 23]. Course of QoL was associated with course of OCD in our study: QoL of patients with remitting OCD improved significantly more than patients with chronic OCD in the first 2 years; this difference in improvement was moderate. Moreover, QoL of chronic patients was overall poorer than QoL of other patients. Our findings are congruent with research findings that QoL is negatively related to OCD symptom severity [19, 22] and in contrast to findings that there is no or only a modest correlation between them [16, 21, 24].

In patients with remitting OCD, more severe comorbid anxiety and depression symptoms were associated with a lower QoL confirming results from previous cross-sectional studies and a previous longitudinal study, in which depressive symptoms predicted QoL at 1-year follow-up [16, 23]. Our findings suggest that comorbid anxiety and depression symptoms were more important for QoL than residual subsyndromal OCD symptoms, implicating that anxiety and depressive symptoms should be given more attention in the treatment of patients with OCD.

Of the sociodemographic variables, only unemployment was associated with an unfavourable QoL in remitting patients. This finding might reflect that having a job contributes to a good QoL. Of the psychosocial variables, less emotional stability was associated with an unfavourable QoL and masked the effect of social network, emotional and social loneliness, and emotional and social support. Possibly, emotionally instable patients are less able dealing with difficulties arising from OCD. The remaining personality traits were not significantly associated with QoL, which is not congruent with a meta-analysis, showing that personality traits and QoL are related [61]. This discrepancy might be explained by a difference in samples: the current study is based on treatment-seeking patients, whereas the meta-analysis is based on convenience samples. Our results might indicate that being extravert, agreeable, conscientious, or autonomous does not help coping with the specific limitations arising from OCD. Severity of anxiety and depression symptoms were more important for QoL than unemployment and emotional instability in our study, which might indicate that feeling anxious or depressed overwhelms limitations caused by unemployment or emotional instability. In previous, cross-sectional studies, contradictory results were found on the effect of employment on QoL and the effect of emotional stability was not studied before [26, 62, 63]. Age, gender, and perceived lack of support were not associated with course of QoL in our study, contrasting results from previous cross-sectional studies [15, 25, 30]. Our study suggests that QoL in remitting patients might be improved by treating comorbid anxiety and depression symptoms, stimulating employment, and by developing more emotional stability, for instance by training emotion regulation.

In the literature, it has been suggested that a low QoL that remains after remission of a mental disorder may be explained by three hypothetical causes: a trait effect (the low QoL was already present before the onset of the disorder), a scar effect (the low QoL is the result of the disorder), or a state effect (the low QoL is the result of residual mental or somatic symptoms [13]). Although our results suggest a state effect (compared with the normal population, the lower QoL in remitters from OCD is caused by remaining comorbid anxiety and depression symptoms) we cannot rule out that the former presence of OCD has induced a so-called ‘scar’, since we do not have data on the QoL before the onset of the OCD. A scar effect might be manifest in, for example, unemployment, a small social network, or not having a partner or a satisfying family life. These scar effects can reinforce each other and further deteriorate QoL; for example, OCD can cause low social status by impairing educational achievement and ability to work, and can reduce social support by either alienating family members or by decreasing the likelihood of finding a partner.

Strengths of this study are that we had access to a large, representative sample of treatment-seeking patients with OCD who were followed for a long period of time. Thus, our results are generalizable to clinically referred OCD patients in a specialized setting [36]. Furthermore, this is the first longitudinal study that examined a broad range of variables possibly associated with course of QoL in patients with remitting OCD; however, future research should examine whether QoL can indeed be improved by targeting the characteristics that were significantly associated with QoL. A limitation of this study is that predictors that are thought to be relatively stable over time (partner, children, education, employment, and expressed emotion) were measured at baseline only. The effect of a possible change of these predictors on QoL at 2-year and 4-year follow-up is missing. In addition, a certain amount of overlap between OCD and diminished QoL is to be expected, because the DSM diagnosis of obsessive–compulsive disorder incorporates psychosocial dysfunctioning. Likewise, an overlap between anxiety/depression and QoL is not surprising either as psychosocial health is an important aspect of QoL. Nevertheless, our finding that depression and anxiety symptoms are more important for QoL than residual OCD symptoms is important as it underlines the potential opportunity to improve QoL by focusing on depression and anxiety. Furthermore, QoL, as defined by the WHO, encompasses six dimensions: physical health, psychological health, level of independence, social relationships, environment, and spirituality. The EQ-5D does not encompass all of these dimensions. Despite this, the EQ-5D is frequently used and regarded a valid and reliable instrument to assess QoL [40–43]. Finally, the group of patients with an intermittent OCD was small, so results on this group need to be verified in future studies.

Historically, clinical research took symptom reduction as an outcome measure with the implicit assumption that patients would be able to resume their life when the disorder was remitted. However, it appears that the relationship between clinical symptoms and QoL is only moderate and remission of symptoms is not sufficient for restoring QoL [64, 65]. Moreover, patients place more importance on their ability to live a fulfilling life than on absence of symptoms [66]. Nowadays, the importance of taking a broader view on the life of patients and not merely on symptoms is widely accepted. This view is congruent with the definition of mental health of the WHO: “a state of well-being in which the individual realizes his or her own abilities, can cope with the normal stresses of life, can work productively and fruitfully, and is able to make a contribution to his or her community” [67]. With QoL outcome measures reflecting QoL aspects such as return to home, work or school, enjoying relationships with family and friends and having a sense of well-being, the perspective of the patient is better taken into account and treatments can be sought that achieve greater effect in these life domains.

Our study indicates that QoL improves in those who remit from OCD, underlining the importance of reducing OCD symptoms in treatment—as an end in itself but also to improve QoL. However, even in remitting patients, QoL may remain impaired, suggesting that clinicians should not only focus on remission of obsessive compulsive symptoms in treatment, but also on QoL of their patients as well.

## Electronic supplementary material

Below is the link to the electronic supplementary material.

Supplementary material 1 (DOCX 53 kb)

## Footnotes

1 Remitting patients had as much education as chronic patients in the multiple imputation data set.

2 Less informative support was significantly related to an unfavourable course of QoL in the univariable analysis on the multiple imputation data set.
