# Supplementary material for: Four-year course of quality of life and obsessive–compulsive disorder
Source: Soc Psychiatry Psychiatr Epidemiol. 2019 Sep 20;55(8):989–1000. doi: 10.1007/s00127-019-01779-7 (PMC7395050; doi:10.1007/s00127-019-01779-7)
Supplement: Supplementary file 1 — Supplementary material 1 (DOCX 53 kb) [file 127_2019_1779_MOESM1_ESM.docx]

**Electronic Supplement**

**Four-year course of quality of life and obsessive-compulsive disorder.**

**Social Psychiatry and Psychiatric Epidemiology**

Karin C.P. Remmerswaal¹, MSc

Neeltje M. Batelaan¹, MD, PhD

Adriaan W. Hoogendoorn¹, PhD

Nic J.A. van der Wee², MD, PhD

Patricia van Oppen¹, PhD

Anton J.L.M. van Balkom¹, MD, PhD

¹ Amsterdam UMC, Vrije Universiteit Amsterdam, Department of Psychiatry and the Amsterdam Public Health Institute, and GGZ inGeest, Amsterdam, The Netherlands.

² Department of Psychiatry, Leiden University Medical Centre, The Netherlands.

Corresponding author: Karin C.P. Remmerswaal, GGZ inGeest, Amstelveenseweg 589, 1081 JC Amsterdam, The Netherlands, k.remmerswaal@ggzingeest.nl, telephone number 0031.20.7885000, fax number 0031.20.3016997

**Content**

**Table 0.** Number of missing observations for all variables in the analyses (n=382)…………………………………………..3

**Table 1.** Baseline characteristics of OCD patients, multiple imputations dataset……………………………………………..4

**Table 2.** Four-year course of QoL of patients with OCD and comparison with QoL of the general population with one sample t-tests¹, multiple imputations dataset, n=382………………………………………………………………………..5

**Fig. 1.** Four-year course of QoL of patients with chronic, intermittent and remitting OCD, multiple imputations dataset, n=382………………………………………………………………………………………………………………………………………………….6

**Table 3.** Results of LMM analysis of the four-year course of QoL of patients with chronic, intermittent and remitting OCD, multiple imputations dataset, n=382……………………………………………………………………………………….7

**Table 4.** Predictors of the four-year course of QoL in remitters from OCD, multiple imputations dataset, n=115.3……………………………………………………………………………………………………………………………………………………………8

**Table 0.** Number of missing observations for all variables in the analyses (*n*=382)

|  | Baseline | 2-year follow-up | 4-year follow-up |
| --- | --- | --- | --- |
| **Baseline characteristics** |  |  |  |
| *Sociodemographic characteristics* |  |  |  |
| Age | 0 |  |  |
| Gender | 0 |  |  |
| Partner | 9 |  |  |
| Children | 0 |  |  |
| Education | 1 |  |  |
| Employment | 0 |  |  |
|  |  |  |  |
| *Clinical characteristics* |  |  |  |
| Age of onset OCD | 39 |  |  |
|  |  |  |  |
| *Psychosocial characteristics* |  |  |  |
| Attachment style: dismissing | 29 |  |  |
| Attachment style: preoccupied | 31 |  |  |
| Attachment style: fearful | 29 |  |  |
| Attachment style: secure | 32 |  |  |
| FFPI¹ extraversion | 19 |  |  |
| FFPI¹ agreeableness | 19 |  |  |
| FFPI¹ conscientiousness | 19 |  |  |
| FFPI¹ emotional stability | 19 |  |  |
| FFPI¹ autonomy | 19 |  |  |
| LEE² lack of emotional support | 20 |  |  |
| LEE² perceived intrusiveness | 20 |  |  |
| LEE² perceived irritation | 20 |  |  |
| LEE² perceived criticism | 20 |  |  |
|  |  |  |  |
| **Time varying characteristics** |  |  |  |
| *Clinical characteristics* |  |  |  |
| Current diagnosis OCD | 0 | 104 | 114 |
| Y-BOCS | 5 | 104 | 115 |
| Number of disorders³ | 0 | 104 | 114 |
| Beck Anxiety Index | 20 | 136 | 138 |
| Beck Depression Inventory | 21 | 134 | 136 |
|  |  |  |  |
| *Psychosocial characteristics* |  |  |  |
| Quality of Life | 23 | 135 | 137 |
| Social network | 9 | 109 | 119 |
| Loneliness emotional | 19 | 134 | 137 |
| Loneliness social | 18 | 134 | 137 |
| Need for affiliation | 22 | 134 | 140 |
| SSI⁴ emotional support | 19 | 137 | 136 |
| SSI⁴ informative support | 19 | 138 | 136 |
| SSI⁴ social companionship | 19 | 137 | 136 |
| SSI⁴ instrumental support | 22 | 139 | 139 |

¹ Five-Factor Personality Inventory

² Level of Expressed Emotion

³ Number of current comorbid psychiatric disorders

⁴ Social Support Inventory

**Table 1.** Baseline characteristics of OCD patients, multiple imputations dataset

|  | **Total sample**  *mean (se)* or %  *n*=382 | *n* |  | **Chronic (1)**  *mean (se)* or %  *n*= 222.6¹ | **Intermittent (2)**  *mean (se)* or %  *n*=44.1¹ | **Remitting (3)**  *mean (se)* or %  *n*=115.3¹ | test statistic | *p*-value | post-hoc  analysis  *p*<0.05 |
| --- | --- | --- | --- | --- | --- | --- | --- | --- | --- |
| *Sociodemographics* |  |  |  |  |  |  |  |  |  |
| Age, years | 36.4 (0.56) | 382 |  | 37.0 (0.78) | 36.5 (2.02) | 35.1 (1.12) | *F*(2,319.2)=0.80 | 0.45 |  |
| Gender, female | 57% | 382 |  | 60% | 55% | 50% | *F*(2,335.2)=1.33 | 0.27 |  |
| Partner, yes | 61% | 382 |  | 58% | 72% | 63% | *F*(2,304.5)=0.95 | 0.39 |  |
| Children, yes | 36% | 382 |  | 36% | 34% | 37% | *F*(2,323.9)=0.03 | 0.97 |  |
| Education, years | 12.6 (0.16) | 382 |  | 12.2 (0.23) | 12.6 (0.58) | 13.1 (0.34) | *F*(2,322.0)=2.17 | 0.12 |  |
| Employment, yes | 52% | 382 |  | 47% | 49% | 63% | *F*(2,331.8)=3.25 | 0.04* | 1<3 |
|  |  |  |  |  |  |  |  |  |  |
| *Clinical characteristics* |  |  |  |  |  |  |  |  |  |
| Y-BOCS total | 21.2 (0.36) | 382 |  | 22.5 (0.50) | 20.2 (1.27) | 19.2 (0.74) | *F*(2,310.1)=5.98 | <0.01* | 1>3 |
| Late age of onset OCD², yes | 38% | 382 |  | 33% | 43% | 46% | *F*(2,312.7)=2.20 | 0.11 |  |
| Number of disorders³ | 1.9 (0.06) | 382 |  | 1.9 (0.08) | 1.9 (0.21) | 2.0 (0.12) | *F*(2,317.0)=0.04 | 0.96 |  |
| Beck Anxiety Index | 18.2 (0.62) | 382 |  | 18.2 (0.90) | 18.2 (2.31) | 18.3 (1.30) | *F*(2,292.4)=0.00 | 1.00 |  |
| Beck Depression Inventory | 16.1 (0.51) | 382 |  | 16.1 (0.74) | 16.1 (1.89) | 15.8 (1.05) | *F*(2,300.4)=0.02 | 0.98 |  |

¹ sub-sample sizes differ across imputed data sets and were averaged

² Onset >= 20 years

³ Number of current comorbid psychiatric disorders

**p*<0.05

**Table 2.** Four-year course of QoL of patients with OCD and comparison with QoL of the general population with one sample t-tests¹, multiple imputations dataset, *n*=382

| EQ-5D | **Baseline**  *mean (SE)*  *n*  *t(df)*  *p*  *ES* | **2-year follow up**  *mean (SE)*  *n*  *t(df)*  *p*  *ES* | **4-year follow up**  *mean (SE)*  *n*  *t(df)*  *p*  *ES* |
| --- | --- | --- | --- |
| Total sample | 0.67 (0.01)  382  t(28856.1)=-16.36  <0.01*  0.92 | 0.76 (0.02)  382  t(1316.5)=-8.93  <0.01*  0.54 | 0.73 (0.01)  382  t(1687.2)=-10.83  <0.01*  0.67 |
| Chronic OCD | 0.66 (0.02)  222.6  t(2596.2)=-12.29  <0.01*  0.96 | 0.71 (0.02)  222.6  t(1443.4)=-9.37  <0.01*  0.75 | 0.68 (0.02)  222.6  t(1285.3)=-10.95  <0.01*  0.88 |
| Intermittent OCD | 0.70 (0.04)  44.1  t(1264.0)=15.92  <0.01*  0.93 | 0.82 (0.04)  44.1  t(1129.3)=-1.49  0.14  0.34 | 0.79 (0.05)  44.1  t(624.8)=-2.17  0.03*  0.49 |
| Remitting OCD | 0.68 (0.03)  115.3  t(1895.4)=26.08  <0.01*  0.84 | 0.82 (0.03)  115.3  t(1929.9)=-2.79  0.01*  0.28 | 0.81 (0.03)  115.3  t(1530.9)=-2.98  <0.01*  0.32 |

¹ one sample t-test against test value 0.89 for the general population

² *ES*= within-time between-group Effect Size of OCD group versus general population (Cohen’s *d*), obtained using pooled standard deviations assuming *SD*=0.20 for the general population

* *p*<0.05

**Fig. 1.** Four-year course of QoL of patients with chronic, intermittent and remitting OCD, multiple imputations dataset, *n*=382.

QoL of patients with remitting OCD improved significantly more than patients with chronic OCD from baseline to two-year follow up (*β(S.E.)*=0.089 (0.036), *p*=0.01). QoL of patients with chronic OCD was significantly worse than the other two groups on average over time (chronic versus remitting: (*β(S.E)*=-0.086 (0.025), *p*<0.01; chronic versus intermittent: (*β(S.E.)*=-0.086 (0.039), *p*=0.01).

The reference line displays the QoL of the general population (0.89)^45^.

**Table 3.** Results of LMM analysis of the four-year course of QoL of patients with chronic, intermittent and remitting OCD, multiple imputations dataset, *n*=382

| EQ-5D | **From Baseline to 2-year follow up**  *β (S.E.)*  *p*  95% *CI*  *ES* | **From 2-year to 4-year follow up**  *β (S.E.)*  *p*  95% *CI*  *ES* |
| --- | --- | --- |
| Chronic versus intermittent patients | -0.078 (0.051)  0.13  (-0.178, 0.023)  -0.32 | -0.005 (0.053)  0.92  (-0.109, 0.099)  -0.02 |
| Chronic versus remitting patients | -0.089 (0.036)  0.01*  (-0.159, -0.019)  -0.31 | 0.026 (0.036)  0.47  (-0.044, 0.097)  0.12 |
| Intermittent versus remitting patients | -0.012 (0.057)  0.84  (-0.123, 0.100)  -0.05 | -0.032 (0.055)  0.56  (-0.139, 0.075)  -0.15 |

*ES* = Between-group between-time Effect Sizes (standardized using pooled start-time standard deviations)

* *p*<0.05

**Table 4.** Predictors of the four-year course of QoL in remitters from OCD, multiple imputations dataset, *n*=115.3

|  | Bivariate | | Model 1 | | Model 2 | | Model 3 | | Model 4 | |
| --- | --- | --- | --- | --- | --- | --- | --- | --- | --- | --- |
|  | *β (S.E.)* | *p* | *β (S.E.)* | *p* | *β (S.E.)* | *p* | *β (S.E.)* | *p* | *β (S.E.)* | *p* |
| *Sociodemographics* |  |  |  |  |  |  |  |  |  |  |
| Age, years | -0.002 (0.002) | 0.45 |  |  |  |  |  |  |  |  |
| Gender, female | 0.005 (0.041) | 0.91 |  |  |  |  |  |  |  |  |
| Partner, yes | -0.032 (0.042) | 0.45 |  |  |  |  |  |  |  |  |
| Children, yes | 0.000 (0.043) | 1.00 |  |  |  |  |  |  |  |  |
| Education, years | 0.008 (0.006) | 0.19 |  |  |  |  |  |  |  |  |
| Employment, yes | 0.100 (0.041) | 0.02* | 0.100 (0.041) | 0.02* |  |  |  |  | 0.030 (0.032) | 0.35 |
|  |  |  |  |  |  |  |  |  |  |  |
| *Clinical characteristics* |  |  |  |  |  |  |  |  |  |  |
| Y-BOCS¹ | -0.007 (0.002) | <0.01* |  |  | -0.001 (0.002) | 0.64 |  |  |  |  |
| Age of onset OCD², late | 0.015 (0.042) | 0.72 |  |  |  |  |  |  |  |  |
| Time of remission³, late | -0.088 (0.041) | 0.03* |  |  | -0.035 (0.033) | 0.30 |  |  |  |  |
| Number of disorders¹ ⁴ | -0.071 (0.017) | <0.01* |  |  | -0.007 (0.016) | 0.67 |  |  |  |  |
| Beck Anxiety Index¹ | -0.012 (0.002) | <0.01* |  |  | -0.006 (0.002) | <0.01* |  |  | -0.006 (0.002) | <0.01* |
| Beck Depression Inventory¹ | -0.016 (0.002) | <0.01* |  |  | -0.011 (0.002) | <0.01* |  |  | -0.011 (0.002) | <0.01* |
|  |  |  |  |  |  |  |  |  |  |  |
| *Psychosocial variables* |  |  |  |  |  |  |  |  |  |  |
| Attachment style: dismissing | 0.008 (0.012) | 0.51 |  |  |  |  |  |  |  |  |
| Attachment style: preoccupied | -0.007 (0.012) | 0.58 |  |  |  |  |  |  |  |  |
| Attachment style: fearful | -0.016 (0.010) | 0.10 |  |  |  |  |  |  |  |  |
| Attachment style: secure | 0.015 (0.013) | 0.24 |  |  |  |  |  |  |  |  |
| FFPI⁵ extraversion | 0.028 (0.017) | 0.11 |  |  |  |  |  |  |  |  |
| FFPI agreeableness | 0.009 (0.017) | 0.60 |  |  |  |  |  |  |  |  |
| FFPI conscientiousness | 0.012 (0.017) | 0.48 |  |  |  |  |  |  |  |  |
| FFPI emotional stability | 0.063 (0.015) | <0.01* |  |  |  |  | 0.051 (0.015) | <0.01* | 0.008 (0.013) | 0.55 |
| FFPI autonomy | 0.014 (0.019) | 0.45 |  |  |  |  |  |  |  |  |
| LEE⁶ lack of emotional support | -0.001 (0.002) | 0.73 |  |  |  |  |  |  |  |  |
| LEE perceived intrusiveness | -0.005 (0.004) | 0.18 |  |  |  |  |  |  |  |  |
| LEE perceived irritation | -0.002 (0.005) | 0.68 |  |  |  |  |  |  |  |  |
| LEE perceived criticism | -0.006 (0.008) | 0.48 |  |  |  |  |  |  |  |  |
| Social network¹ | 0.007 (0.004) | 0.04* |  |  |  |  | 0.005 (0.004) | 0.17 |  |  |
| Loneliness emotional¹ | -0.031 (0.008) | <0.01* |  |  |  |  | -0.018 (0.010) | 0.06 |  |  |
| Loneliness social¹ | -0.021 (0.010) | 0.03* |  |  |  |  | 0.000 (0.011) | 1.00 |  |  |
| Need for affiliation¹ | 0.009 (0.010) | 0.36 |  |  |  |  |  |  |  |  |
| SSI⁷ emotional support¹ | 0.018 (0.007) | 0.01* |  |  |  |  | 0.006 (0.009) | 0.51 |  |  |
| SSI informative support¹ | 0.018 (0.008) | 0.03* |  |  |  |  | 0.002 (0.010) | 0.81 |  |  |
| SSI social companionship¹ | 0.015 (0.006) | 0.02* |  |  |  |  | 0.001 (0.008) | 0.91 |  |  |
| SSI instrumental support¹ | 0.014 (0.007) | 0.06 |  |  |  |  |  |  |  |  |

¹ Time-dependent variable (repeatedly measured)

² Late onset >= 20 years

³ Late remission (at four-year follow up; *n*=31) vs early remission (at two-year follow up; *n*=42)

⁴ Number of current comorbid psychiatric disorders

⁵ Five-Factor Personality Inventory

⁶ Level of Expressed Emotion

⁷ Social Support Inventory

**p*<0.05
